# Incidence of anal incontinence among patients with anal fissure treated with Botox injection versus lateral sphincterotomy

**DOI:** 10.1016/j.amsu.2021.102636

**Published:** 2021-07-29

**Authors:** Rashid Ibrahim, Sabry Abounozha, Ali Yasen Y. Mohamedahmed, Awad Alawad, Ahmed Abdel Rahim

**Affiliations:** a University Hospitals Plymouth NHS Trust, Plymouth, UK; b Northumbria Healthcare NHS Foundation Trust, Northumbria, UK; c Sandwell and West Birmingham NHS Trust, UK; d University Hospital of Wales, Cardiff, UK

**Keywords:** Anal fissure, Botox, Incontinence, Lateral sphincterotomy

## Abstract

A best evidence topic has been constructed using a described protocol. The three-part question addressed was: In patients with anal fissure, which technique has a lower of incidence anal incontinence: Botox injection or lateral sphincterotomy? The best evidence showed that Botox injection has lower incidence of incontinence.

## Highlights

• In patients with anal fissure.
• Which technique has a lower of incidence anal incontinence:
• Botox injection or lateral sphincterotomy?
• Botox injection has lower incidence of incontinence.

## 1 Introduction

This BET was designed using a framework outlined by the International Journal of Surgery [1]. This format was used because a preliminary literature search suggested that the available evidence is of insufficient quality to perform a meaningful meta-analysis. A BET provides evidence-based answers to common clinical questions, using a systematic approach of reviewing the literature.

## 2 Clinical scenario

A general surgical trainee is consenting a 25 year old female with recurrent anal fissure for examination under anaesthesia plus either Botox injection or lateral sphincterotomy, the patient is wondering which technique provides a lower incidence of incontinence.

## 3 Three-part question

[In patient with anal fissure] [Which techniques has lower incidence of incontinence] [Botox injection or lateral sphincterotomy]?

## 4 Search strategy

A. Medline ® 1946 to May 2021 and Embase 1974 to May 2021 using OVID interface:

[Anal fissure OR fissure-in-ano] AND [incontinence OR anal incontinence] AND [botulinum toxin OR botulinum toxin injection OR BOTOX] AND [sphincterotomy OR lateral sphincterotomy OR lateral internal sphincterotomy].

B. Medline ® using PubMed interface:

[Anal fissure OR fissure-in-ano] AND [incontinence OR anal incontinence] AND [botulinum toxin OR botulinum toxin injection OR BOTOX] AND [sphincterotomy OR lateral sphincterotomy OR lateral internal Sphincterotomy].

Exclusion criteria:

Unpublished studies, case reports, letter to the editors, studies in children less than 16 years studies not in English.

## 5 Search outcome

A total of 67 articles were identified after the removal of duplicates. Of these 51articles were excluded on the basis of title and abstract. After full-text assessment of the remaining 16 articles another 11 articles were excluded because they did not include the information needed to answer the question. A total of 5 articles (3 randomized controlled trials, one prospective and one retrospective studies) were identified to provide the best evidence to answer the question.

## 6 Result

see the Table 1.

**Table 1:** Result.

Author, **date of publication, journal name and country**		Study type **and level of evidence**	Patient group& Dose of Botox	Outcomes **Follow up**	Key results	Additional **comments**
De Robles et al. 2021 Asian Journal of Surgery Australia		Retrospective study level III	Total number of patients 251 **Group1 (BT):** 81 **Group2 (LIS):** 171 Dose of Botox 30IU	Primary endpoint: Incidence of anal incontinence The mean follow- up period was 5 years (range 1–10 years).	Long-term incontinence **Group1** = 0 (0 %) **Group2** = 4 (1.5 %) P = 0.317	-Single centre, -large sample size, -long period of follow up -retrospective analysis
					Difference is not statistically significant	
Çakır et al. Turk J Surg 2020 Turkey		Retrospective study level III	A total of 135patients: **Group1 (BT): 61** **Group2 (LIS): 74** Dose of Botox 50IU	Primary endpoint: Incidence of anal incontinence follow-up 1 year.	**Group1** = 0 (0 %) **Group2** = 2 (2.7 %) (p = 0.290)	-multi centre, -large sample size, -Retrospective
					Difference is not statistically significant	
Sebastián et al. Med Clin (Barc). 2005 Spain		prospective randomized trial level II	Total number of patients 80 Group1 (BT): 40 Group2 (LIS): 40 Dose of Botox 25 IU	Primary endpoint: Incidence of anal incontinence The mean follow- up period was 3-year	Group1: 0 (0 %) Group2: 2 (5%) P = 0.05 Difference is statistically significant	Single centre, -Small sample size, -Short period of follow up -only patient with closed internal lateral sphinctrotomy were include
Nasr et al. World J Surg (2010) Egypt	Randomized, Controlled Trial Level II		Total number of patients 80 Group1 (BT): 40 Group2 (LIS): 40 Dose of Botox 25 IU	Primary endpoint: Incidence of anal incontinence median follow-up 18 weeks.	Group1: 0 (0 %) Group2: 6 (51 %) (P = 0.0338). Difference is statistically significant	-Single centre, -Small sample size, -Short period of follow up
Valizadeh et al. Langenbecks Arch Surg 2012 Iran	Randomized, Controlled Trial Level II		Total number of patients 80 Group1 (BT): 40 Group2 (LIS): 40 Dose of Botox 50IU	Primary endpoint: Incidence of anal incontinence follow-up 1 year.	**Group 1** = 0 (0%) **Group 2=** 1 (4%) (P = 0.05). Difference is statistically significant	-Single centre, -Small sample size

## 7 Discussion

Lateral internal sphincterotomy (LIS) is usually performed by creating a vertical incision in the intersphincteric groove on one side of the anus, the internal sphincter fibers are then divided up to the level of the proximal extent of the anal fissure [2]. LIS has been reported as the procedure of choice for anal fissure that is not responding to conservative treatment [3]. However, one of the main drawback of this procedure is potential anal incontinence [4]. Since Botox was introduced as a potential treatment for anal fissures [5], many studies have suggested promising results with lower complications rate. The main advantage of using Botox in comparison to sphinctrotomy is that Botox decreases the anal resting tone, promoting fissure healing, without permanent damages to anal sphincters [6]. Anal incontinence is defined as the involuntary loss of gas, liquid or faeces persisting at the 12-month of follow up [3].

The aim from this review is to assess the best studies which compare the incidence of anal incontinence among those patients undergoing Botox injection vs LIS for anal fissure.

Two studies in our review showed no statistically significant difference in the incidence of anal incontinence between LIS and Botox injection these studies were conducted by De Robles et al. [7] and Çakır et al. [8]. Although both study included relatively large sample size, they are lacking randomization. In contrast, the other three studies we have included, which are all randomized control trials showed a statistically significant lower of incidence of anal incontinence among the Botox injection group in comparison to the LIS group [[9], [10], [11]], the only limitations in these trials is lack of multicentricity, relatively small sample size.

## 8 Clinical bottom line

According to the above articles, the best evidence showed a statistically significant lower incidence of anal incontinence among Botox injection group of patients in comparison to the LIS group.

## 9 Limitation of this review

1. Small sample size in most articles
2. Short period of follow in most articles.
3. Lack of multicentric trials

## Sources of funding

Non.

## Ethical approval

Not applicable.

## Consent

Not applicable.

## Authors contribution

RI: conducted the literature search and wrote the paper. AAR: assisted in the literature search and SA: assisted in writing of paper.

AA: assisted in the literature search.

AM: design the table and help in editing of writing.

## Trial registry number

Not applicable.

## Guarantor

Rashid Ibrahim.

## Declaration of competing interest

Non.
